# Intranasal L-MYC Neural Stem Cells Restore Neurovascular and Neuroimmune Homeostasis After TBI via CX3CL1 Upregulation and ICAM-1 Downregulation

**DOI:** 10.64898/2026.10.09.756809

**Published:** 2026-10-12

**Authors:** Brecken Enright, Jeffrey P. Cheng, Vikram Adhikarla, Isabella Vasquez, Yate-Ching Yuan, Corina O. Bondi, Russell Rockne, Anthony E. Kline, Margarita Gutova

## Abstract

Traumatic brain injury (TBI) triggers persistent neuroimmune and neurovascular dysfunction that contributes to secondary brain injury and limits recovery. Here, we investigated whether a single intranasal dose of LMNSC01 could reprogram post-traumatic neuroinflammatory and neurovascular responses and whether these effects were conserved across biological sex. Immunocompetent male and female rats underwent a controlled cortical impact (CCI) or Sham surgery and 7 days later received a single bolus dose of LMNSC01 (6 × 10 ^6^ cells/dose) or vehicle. Integrated histological and transcriptomic analyses and multiplex tissue ELISA were used to characterize injury- and treatment-associated responses.

CCI induced persistent but sex-dependent transcriptional changes. Male rats exhibited prominent innate immune, adhesion, extracellular matrix, and tissue remodeling responses, whereas females demonstrated broader activation of microglial, interferon, complement, MHC/antigen presentation, and humoral/adaptive immune pathways. LMNSC01 treatment reprogrammed these distinct injury states through partially sex-specific mechanisms. In males, LMNSC01 increased endogenous immune-regulatory mediators, including *Il1rn* , and reduced pathways associated with adhesion and immune-cell trafficking. In females, treatment decreased expression of genes related to microglial, leukocyte, MHC/antigen-presentation, cytokine, and adhesion pathways, and reduced macrophage- and CD45-positive cell signatures. Despite these sex-dependent transcriptional changes, LMNSC01 produced conserved protein changes in both sexes, reducing injury-associated ICAM-1 and restoring CX3CL1 proteins, proposing a novel molecular mechanism of LMNSCs modulating endothelial activation and neuron– microglia communication post injury. Our study provides further insight into sex differences in responses to injury and treatment after TBI and highlights candidate repair mechanisms, supporting further development of LMNSC01 as a minimally invasive therapy for TBI.

**Significance Statement:**

This study provides evidence that a single intranasal dose of LMNSC01 can reprogram chronic neuroimmune and neurovascular dysfunction after TBI in both sexes. Despite distinct male and female injury and treatment responses, LMNSC01 produced a conserved reduction in ICAM-1 and increase in CX3CL1, suggesting reduced endothelial activation and restoration of homeostatic neuron–microglia signaling. Identification of shared changes in neurovascular and neuroimmune signaling provides a candidate mechanism for neural stem cell-mediated repair and supports the translational development of non-invasive intranasal LMNSC01 therapy for TBI.

